# Long-term outcomes of IVUS-guided and angiography-guided drug-eluting stent implantation for left main coronary artery disease: a retrospective consort study

**DOI:** 10.1186/s13019-024-02800-0

**Published:** 2024-07-01

**Authors:** Qing Yang, Xiaoyi Ma, Yuexi Wang

**Affiliations:** 1 Department of Emergency Cardiovascular Medicine, Inner Mongolian People Hospital, Hohhot, 010000 China; 2 Department of cardiovascular medicine, Dalat people’s Hospital, Erdos, 014300 China; 3 Department of cardiovascular medicine, Affiliated Hospital of Inner Mongolia Medical University, No. 1, Tongdao North Road, Hohhot, 010000 Inner Mongolia Autonomous Region China

**Keywords:** Unprotected left main coronary artery disease, Intravascular ultrasound, Left main coronary artery, Drug-eluting stent, Adverse cardiac events, Minimum stent diameter

## Abstract

**Background:**

In patients with unprotected left main coronary artery disease (ULMCAD), this study compared the long-term prognosis of drug-eluting stent insertion guided by intravascular ultrasonography (IVUS) vs. angiography.

**Patients and methods:**

This retrospective consort investigation was performed in December 2021. This analysis included 199 patients who underwent IVUS-guided (IVUS group, *n* = 81) or angiography-guided (angiography group, *n* = 118) drug-eluting stent implantation at the Affiliated Hospital of Inner Mongolia Medical University between September 2013 and September 2018. Major adverse cardiac events (MACE) were defined as cardiovascular death, sudden cardiac death, myocardial infarction.

**Results:**

The IVUS group had considerably lower proportions of MACE within 1 year postoperatively (*P* = 0.002) and cardiac mortality within 3 years postoperatively (*P* = 0.018) compared to the angiography group. However, after adjusting for confounding variables, the hazard ratio for 3-year cardiac mortality was similar between the two groups (*P* = 0.28). In the IVUS group, there was considerably greater minimum lumen diameter (MLD) (*P* = 0.046), and reduced frequencies of target vessel restenosis (*P* < 0.050) and myocardial infarction (MI) (*P* = 0.024) compared to the angiography group. Cox regression analysis for 3-year cardiac mortality found that MSD was independently associated with low cardiac mortality (HR = 0.1, 95% CI: 0.01–14.92, *P* = 0.030).

**Conclusion:**

IVUS-guided drug-eluting stent implantation may lead to better long-term prognosis in patients with ULMCAD, and MSD may be a predictor for lower cardiac mortality.

## Introduction

Globally, coronary artery disease is a leading cause of illness and mortality. Percutaneous coronary intervention (PCI) is the primary therapeutic intervention for this condition [1]. Left main coronary artery disease (LMCAD) is a severe form of ischemic heart disease that poses a considerable risk of mortality and poorer clinical outcomes compared to other patients with ischemic heart disease. PCI was previously contraindicated for LMCAD, but with advances in drug-eluting stents (DES) and imaging techniques, it has become a more commonly used treatment option [2, 3].

Coronary angiography provides only a two-dimensional view of the vessel lumen and does not offer information about plaque burden or stent apposition. Angiography-guided stent implantation (SI) relies on visual estimation of the vessel size and lesion length, which may result in stent undersizing or oversizing, leading to stent malapposition and incomplete lesion coverage, which is inadequate for assessing complex lesions such as LMCAD [4]. Intravascular ultrasound (IVUS) and optical coherence tomography (OCT) are advanced intracoronary imaging techniques used to provide detailed 3D morphological information of coronary plaques, vessels, and stents. Studies have demonstrated that SI guided by IVUS can result in improved outcomes compared to angiography-guided implantation [5]. IVUS enables high-resolution, cross-sectional images of the coronary artery, allowing for precise assessment of the vessel lumen, plaque burden, and stent placement [6, 7]. By identifying stent malapposition, which is associated with an increased risk of stent thrombosis and restenosis, IVUS-guided SI can lead to better stent apposition and expansion, reducing the risk of adverse events. In addition, the decreased incidences of severe adverse cardiovascular events like death, myocardial infarction, and target lesion revascularization have been associated with IVUS-guided stenting [8–10]. These results make it possible to advise ideal SI with more thorough information [11, 12]. The effects of IVUS-guided stenting on coronary imaging findings before and after SI have been studied in a number of randomized controlled trials and observational studies [13], which have shown that IVUS-guided stenting is especially helpful for patients with complex coronary artery disease, such as severe calcifications, long lesions, unprotected left main disease, bifurcations, chronic total occlusion, and other complex conditions [8].

Despite the growing evidence supporting the use of IVUS to optimize PCI, its use in intracoronary imaging to guide stent placement during re-interventional clinical practice is still low. The lack of supporting clinical evidence and high costs have limited the popularity of this technology [14]. Therefore, we aim to investigate the clinical outcomes of PCI guided by angiography alone versus adjunctive IVUS imaging, as well as the clinical benefits of IVUS-guided SI in left main coronary artery (LMCA) stenosis.

## Methods

### Study design and participants

This study was a retrospective study that was performed on December 2021. This study reviewed the clinical data and follow up results of the patients treated at the Department of Cardiology, the Affiliated Hospital of Inner Mongolia Medical University, during the period from September 2013 to September 2018, in order to assess the effectiveness of PCI in patients with ULMCAD. ULMCAD was defined as stenosis of the LMCA causing at least a 50% reduction in blood vessel diameter [17]. Patients between 60 and 80 years of age with coronary LMCAD who received DES implantation and standard antiplatelet therapy after PCI were included. Patients who had a history of acute myocardial infarction (MI) within 24 h, cardiogenic shock, high risk for bleeding, liver insufficiency, malignant tumor, or had coronary chronic total occlusion (CTO) of left anterior descending (LAD) or left circumflex (LCX) artery and underwent rotational atherectomy within 3 months were excluded. The ethical committee of the Affiliated Hospital of Inner Mongolia Medical University authorized this study, and written informed permission were provided by all participants. All interventional treatments were performed according to the current standards.

### Treatment methods

Four experienced surgeons conducted coronary angiography or IVUS procedures to assist in determining the optimal stenting method for patients, based on the criteria such as vessel diameter, stent expansion status, stent edge adherence, and presence of dissection at the edge. If optimal stenting was suggested with the assistance of IVUS and the patients agreed and underwent stenting with IVUS, then the patients were allocated to the IVUS group. If optimal stenting was suggested with the assistance of coronary angiography and the patients agreed and underwent stenting with coronary angiography, then the patients were allocated to the angiography group. Patients who had suboptimal stenting as determined by angiography or IVUS without further dilation were randomly included in the IVUS and angiography groups based on a random table method. The choice of medication and use of tools such as glycoprotein IIb/IIIa inhibitors, low molecular weight heparin, DES type, preconditioning, and intra-aortic balloon pump were at the discretion of the surgeon. Prior to surgery, patients received a 300 mg loading dose of clopidogrel. Post-dilation was recommended for both groups using a non-compliant balloon with a balloon/stent ratio of 1.0:1.0 as determined by angiography or IVUS. For patients with suboptimal stent expansion or stent malposition, post-expansion was performed as needed. IVUS was performed only if the patient was not at risk of circulatory failure. The effectiveness of stent placement was evaluated immediately after surgery using postoperative IVUS imaging with a Volcano’s s5 IVUS instrument. The ideal results as determined by IVUS were defined as thrombolysis in myocardial infarction (TIMI) blood flow grade 3, a minimum stent lumen cross-sectional area of > 6.9 mm^2^, fully expanded and adherent stent, no blood flow between the stent and vascular intima, and no vascular dissection as noted in references [15, 16]. Angiographic success was defined as TIMI grade 3 with < 10% residual stenosis. Following the procedure, patients were prescribed lifelong aspirin 100 mg/day and clopidogrel 75 mg/day for at least 12 months.

### Data collection and definition

The study collected various baseline clinical characteristics of the patients, including demographic information such as age, gender, height, and weight, as well as comorbidities like hypertension, hyperlipidemia, diabetes, stroke, atrial fibrillation, smoking, chronic renal insufficiency, serum creatinine, history of myocardial infarction, acute myocardial infarction, non-ST-segment elevation myocardial infarction, ST segment elevation myocardial infarction, cardiogenic shock, left ventricular ejection fraction. Lesion features such as left anterior descending (LAD), left circumflex (LCX), right coronary artery (RCA), and LMCAD were also recorded, along with the location of ostial lesion, bifurcation lesion, coronary body, and LMCAD plaque characteristics like calcified lesions and medina classification. The study also collected information on the average number of stents, average implanted stent diameter, and average length of implanted stent, as well as post-expansion cases, synergy between PCI with TAXUS DES and cardiac surgery (SYNTAX) score, Numeric Rating Scale (NERS) score, thrombolysis in myocardial infarction (TIMI) blood flow grade, preoperative minimum lumen area (MLA), preoperative minimum lumen diameter (MLD), plaque burden, and minimum stent inner diameter (MSD). Furthermore, the study collected data on surgical hospitalization, outpatient follow-up, and 3-year follow-up records.

### Outcomes

The primary outcomes of the study were the occurrence of main adverse cardiac events (MACE) at 1- and 3-year intervals. MACE were defined as cardiovascular death, sudden cardiac death, myocardial infarction. Cardiac mortality was considered the cause of all deaths, except in cases where clinical or autopsy findings indicated a non-cardiac origin. The secondary outcomes of the study were the risk of stent thrombosis (RST), and it was then divided into three categories: early (0–30 days after SI), late (31–360 days), and extremely late (> 360 days) [17]. This information was categorized as certain, likely, or possible. The study also evaluated target vessel lesion restenosis (TVR), myocardial infarction (MI), and CABG as secondary outcomes. During the follow up period, CT scan of coronary arteries were performed to check the patients’ status, and angiography was performed when the patients had obvious symptoms and unstable status.

### Statistical analysis

The statistical analysis was performed using Stata version 12.0. Continuous variables were assessed for normality using the Kolmogorov-Smirnov test and reported as mean ± standard deviation or median. Normally distributed variables were compared using the Student’s t-test, while the Mann-Whitney U test was used for skewed variables. Categorical variables were presented as frequencies or percentages and compared using chi-square statistics or Fisher’s exact test. Kaplan-Meier method was used to generate survival curves, and the log-rank test was used to assess differences between them. To identify independent predictors of the primary endpoint, a multivariate Cox proportional hazards regression model was used with relevant variables from univariate analysis (with *P*-values ≤ 0.1) and previous research reports used as covariates. The Grønnesby-Borgan-May test was used to evaluate the goodness of fit of the Cox multivariate model. Hazard ratios, 95% confidence intervals, and *P*-values were reported as findings. A two-tailed *P*-value less than 0.05 was considered statistically significant.

## Results

### Comparison of baseline clinical characteristics between angiography and IVUS groups

Table 1 showed baseline data comparison between the angiography and IVUS groups. The angiography group had 118 patients with mean age of 68.1 ± 8.8 years, while the IVUS group had 81 patients with mean age of 65.6 ± 8.1 years. Age in the angiography group was older than the IVUS group (*P* = 0.042). The prevalence of hypertension and hyperlipidemia in the angiography group was greater than in the IVUS group (*P* < 0.05). The IVUS group had a considerably greater mean implanted stent diameter (3.7 ± 0.6 mm) than the angiography group (3.4 ± 0.2 mm)( *P* < 0.001). The proportion post-expansion cases in IVUS group was considerably higher than that in the angiography group (82.7% vs. 61.0%; *P* < 0.001). The mean minimum stent diameter (MSD) in the IVUS group was considerably larger than in the angiography group (3.8 ± 0.3 mm vs. 3.3 ± 0.3 mm; *P* < 0.001). The other variables were comparable between the two groups (*P* > 0.05).

**Table 1:** Comparisons of baseline clinical characteristics between the angiography group and IVUS group

Variables	Angiography group (*n* = 118)	IVUS group (*n* = 81)	*P* value
Age (Y)	68.1 ± 8.8	65.6 ± 8.1	0.042*
Gender, n (%)			0.090
Males	82(69)	65 (80)	
Females	36 (31)	16 (20)	
Height (cm)	171.9 ± 8.2	171.4 ± 5.9	0.617
Weight (Kg)	75.7 ± 10	73.4 ± 7.6	0.062
Hypertension	93(79)	74 (91)	0.018*
Hyperlipidemia	91(77)	77 (95)	0.001*
Diabetes	96(81)	73 (90)	0.089
Stroke	9(8)	9 (11)	0.400
Atrial fibrillation	11(9)	7 (7)	0.869
Smoking	95(81)	70 (86)	0.276
Chronic renal insufficiency	6(5)	4 (5)	0.963
Serum creatinine, μmol/L	84.1 ± 15.7	85.2 ± 16.6	0.616
History of myocardial infarction	36(31)	23(28)	0.748
ST segment elevation myocardial infarction	1(1)	1 (1)	0.788
Non-ST-segment elevation			
Myocardial infarction	2(2)	0 (0)	0.239
Cardiogenic shock	4(3)	2 (2)	0.709
Left ventricular ejection fraction	63 ± 5.8	63 ± 6	0.839
History of PCI	15(13)	12 (15)	0.670
Lesion features			
LAD	91(77)	52(64)	0.082
LCX	48(41)	40(49)	0.224
RCA	17(14)	20(25)	0.067
LMCAD location			
Ostial lesion	23(19)	23(28)	0.143
Bifurcation lesion	56(47)	34(42)	0.445
Coronary body	39(33)	24(30)	0.61
LMCAD plaque characteristics Calcified lesions	42(36)	33(41)	0.462
Medina classification			
1,0,0	9(8)	2 (3)	0.118
1,1,0	18(15)	15 (19)	0.543
1,0,1	16(14)	18 (22)	0.111
0,1,1	0(0)	7 (9)	0.001
0,1,0	11(9)	7 (9)	0.869
0,0,1	16 (14)	1 (1)	0.002
1,1,1	48 (41)	31 (38)	0.733
Average number of stents	2.0 ± 1.1	2.0 ± 1.0	0.020*
Average implanted stent diameter (mm^2^)	3.4 ± 0.2	3.7 ± 0.6	< 0.001*
Average length of implanted stent (mm)	32.1 ± 20.4	32.4 ± 19.6	0.17
Post-expansion cases	72 (61.0)	69(82.7)	< 0.001*
TIMI blood flow < grade 3, n (%)			
Preoperative	118	81	1.000
Postoperative	0(0)	0(0)	1.000
SYNTAX score	30.6 ± 4.8	30.8 ± 5	0.716
NERS score	26.2 ± 5.5	26.4 ± 5.5	0.822
Preoperative MLA (mm^2^)	4.1 ± 0.2	4.1 ± 0.3	0.725
Preoperative MLD (mm)	1.7 ± 0.3	1.7 ± 0.3	0.724
Plaque burden (%)	74 ± 12.9	73.6 ± 10.6	0.245
MSD (mm)	3.3 ± 0.3	3.8 ± 0.3	< 0.001*

Values are expressed as mean ± standard deviation

IVUS: Intravascular Ultrasound; PCI: percutaneous coronary intervention; LAD: left anterior descending; LCX: left circumflex; RCA: right coronary artery; LMCAD: Left main coronary artery disease; TIMI: thrombolysis in myocardial infarction; MLA: minimum lumen area; MID: minimum lumen diameter; MSD: minimum stent diameter; NERS: numeric Rating Scale

*P < 0.05

### Follow-up outcomes comparison between angiography and IVUS groups

Table 2**shows follow-up outcomes of the patients.** The incidence rate of MACE occurred in one year in the IVUS group was considerably lower than in the angiography group (6.2% vs. 22.9%; *P* = 0.002). The incidence rate of cardiac death in one year was considerably lower in the IVUS group than the angiography group (1.2% vs. 7.6%; *P* = 0.043). Similarly, the lower incidence of cardiac mortality was observed in the IVUS group compared to the angiography group after two and three years (*P* = 0.049 and *P* = 0.018, respectively). In one year, the IVUS group had a lower incidence of MI than the angiography group (6.2% vs. 16.9%; *P* = 0.024).

**Table 2:** Follow-up outcomes comparisons between the angiography group and IVUS group

Variables	Angiography group (*n* = 118)	IVUS group (*n* = 81)	*P* value
MACE occurred in 1 year	27 (22.9)	5 (6.2)	0.002*
Cardiac mortality in 1 year	9 (7.6)	1 (1.2)	0.043*
Cardiac mortality in 2 years	18 (15.3)	5 (6.2)	0.049*
Cardiac mortality in 3 years	27 (22.9)	8 (9.9)	0.018*
MI occurred in 1 year	20 (16.9)	5 (6.2)	0.024*
MI occurred in 2 year	23(19.5)	8(9.9)	0.027*
MI occurred in 3 year	24(20.3)	8(9.9)	0.031*
Target lesion restenosis in 1 year	10 (8.5)	1 (1.2)	0.041*
Target lesion restenosis in 2 years	10 (8.5)	1 (1.2)	0.041*
Target lesion restenosis 3 years	12 (10.2)	2 (2.5)	0.037*
CABG occurred in 1 year	0 (0)	0 (0)	1.000
CABG occurred in 2 year	3(2.5)	1(1.2)	0.923
CABG occurred in 3 year	3(2.5)	1(1.2)	0.923
Risk of stent thrombosis in 1 year	8 (6.7)	1 (1.2)	0.064
Risk of stent thrombosis in 2 year	9(7.6)	1(1.2)	0.056
Risk of stent thrombosis in 3 year	9(7.6)	2(2.5)	0.052
MLD 1 year after surgery	2.9 ± 0.8	3.5 ± 0.5	0.046*

IVUS: Intravascular Ultrasound; MACE: major adverse cardiac events; MI: myocardial infarction; CABG: Coronary Artery Bypass Grafting; MID: minimum lumen diameter. *P < 0.05

Target lesion restenosis occurred more frequently in the angiography group, compared to the IVUS group, in one, two and three years, respectively (*P* = 0.041, *P* = 0.041, *P* = 0.037, respectively). Within one year, neither group had a CABG (*P* = 1.000). The probability of stent thrombosis within one year had similar trend between the angiography and IVUS groups (IVUS: 8 cases, 6.7%; angiography: 1 case, 1.2%; *P* = 0.064). One year following surgery, the MLD in the IVUS group (3.5 ± 0.5) was considerably greater than in the angiography group (2.9 ± 0.8; *P* = 0.046).

### The Cox regression analysis for 3-year cardiac mortality

The results of a Cox proportional hazard regression model study showed that the hazard ratios for 3-year cardiac mortality (HR = 1.89, 95%CI: 0.6-6.0, *P* = 0.280) were comparable between the angiography group and IVUS groups after accounting for average implanted stent diameter, post-expansion cases, MSD, and MLD at 1 year following surgery. However, it was discovered that MSD was independently correlated with 3-year cardiac mortality (HR = 0.1, 95% CI: 0.01–14.92, *P* = 0.030) (Table 3).

**Table 3:** Cox regression analysis for 3-year cardiac mortality

General information	HR, 95%CI	*P* value
IVUS	-	Ref.
Angiography	1.89, 0.6-6.0	0.28
Preoperative MLA (mm^2^)	2.2, 0.4–14.1	0.400
Preoperative MLD(mm)	1.1, 0.1–11.4	1.000
MSD (mm)	0.1, 0.01–14.92	0.030*
MLD at 1 year after PCI	3.4, 0.1–29.5	0.600

IVUS: Intravascular Ultrasound; HR: hazard ratio; MLA: minimum lumen area; MID: minimum lumen diameter; MSD: minimum stent diameter; PCI: percutaneous coronary intervention. *P < 0.05

## Discussion

The age, hypertension, hyperlipidemia, and hyperglycemia were considerably different between the angiography and IVUS groups. There were no considerable differences in gender, height, weight, atrial fibrillation, smoking, renal insufficiency, history of MI, left ventricular ejection fraction, or plaque burden. The MSD was considerably larger in the IVUS group than in the angiography group. Additionally, the study found that MSD may be an independent protective factor against cardiac mortality. From the follow up results, the mortality rate of IVUS group was lower than the angiography group. According to the study’s findings, IVUS-guided SI may offer patients with ULMCAD longer-lasting results than angiography-guided SI in a 3-year period.

With the advent of DES, LMCAD is no longer a contraindication for PCI [18, 19]. A landmark 5-year study of 1905 patients found no considerable difference in combined outcomes of mortality, stroke, or myocardial infarction between PCI and CABG in patients with LMCAD of low or moderate anatomical complexity [18].

Our results showed that angiography was associated with considerably better outcomes in terms of MACE, cardiac mortality, MI, and target lesion restenosis, compared to IVUS. The only variable that did not show a statistically considerable divergence between the angiography and IVUS groups was the risk of stent thrombosis. Additionally, IVUS was associated with a larger minimum lumen diameter after surgery. Overall, the results suggest that IVUS may be a preferable diagnostic tool for coronary artery disease compared to angiography. Because it enables a more precise evaluation of the amount and severity of coronary artery disease, IVUS is an important tool for guiding SI [20]. Compared to other invasive intravascular imaging modalities, High spatial resolution, real-time visualisation of the artery wall, and accurate assessment of stent growth and apposition are only a few benefits of IVUS [21, 22]. IVUS can also reveal whether plaques, calcification, or thrombus are present, which helps doctors choose the right stent size and model for a particular lesion [23]. Therefore, IVUS imaging can be considered an effective tool for guiding SI and improving the outcomes of PCI in patients with ULMCAD.

IVUS-guided treatment may yield better outcomes and lower mortality rate when compared to angiography-only guided treatment according to previous reports [24, 25]. The study did not find a difference in suspected stent thrombosis between the angiography group and IVUS groups. No diversity in postoperative TIMI blood flow was observed between the angiography group and IVUS groups. This suggests that stent thrombosis and TIMI blood flow alone may not be sufficient to evaluate SI, and the condition of the diseased blood vessel itself must be taken into consideration. If necessary, post-expansion or auxiliary stent placement can be performed to ensure that the stent is completely adherent and that no intima falls into the stent, in order to decrease the occurrence of stent thrombosis and the incidence of MACE.

However, it is important to note that the conclusions drawn from this study should be further verified with more case studies. The study highlights the potential of IVUS examination immediately after SI to evaluate its effectiveness. The anatomical grading technique used by IVUS combines circumference (180 degrees) and depth (intima, medium, and adventitia) to identify more anatomical features than angiography. This makes IVUS a valuable tool in predicting patency and complications following SI. Deep anatomical changes may trigger restenosis, while intimal tears may fall into the lumen, leading to acute or subacute thrombotic events, or requiring assisted stenting. The usefulness of IVUS in peripheral arterial studies has already been well established [26].

At the one-year follow-up, our results suggested that there was no considerable difference between the use of angiography and IVUS in terms of hazard ratio. The preoperative MLA and MLD did not considerably affect the choice of diagnostic tool. However, the mean stenosis diameter was considerably higher in the angiography group compared to the IVUS group, which may have influenced the selection of diagnostic tool. The MLD at 1 year post-surgery did not considerably differ between the angiography and IVUS groups. Therefore, the study indicated that the diagnostic tool chosen may not considerably impact post-operative outcomes in patients undergoing coronary artery bypass grafting surgery. But there was a statistically considerable reduction in the occurrence of MACE and cardiac mortality in the IVUS group, as compared to the angiography group.

Therefore, it can be concluded that IVUS guidance is superior to CAG guidance during PCI, regardless of the presence or absence of diabetes, acute coronary syndrome, hypertension, and location of LMCAD. This conclusion is consistent with recent meta-analyses and previous studies [25, 27]. Furthermore, the occurrence of target lesion restenosis and target vessel restenosis was considerably lower in the IVUS group compared to the angiography group. In-stent restenosis was also less frequent in the IVUS group compared to the angiography group, and this difference was statistically considerable. The minimum post-stent lumen area was found to be an independent predictor of the primary endpoint, as confirmed by COX analysis. These findings are consistent with the results of several previous studies [28, 29] and a meta-analysis [30].

The use of IVUS before SI also helps in assessing lipid plaque distribution and collateral access in the distal LMCA. IVUS can also aid in judging the true angle of the distal LMCA bifurcation and thus help determine the optimal PCI strategy. Additionally, IVUS guidance enables the selection of appropriate stent diameters and lengths, optimizing SI procedures for better clinical outcomes [31]. The use of IVUS guidance during SI has been shown to be a superior method for the treatment of left main coronary artery disease (LMCAD), according to a recent study. However, the study also had some limitations that should be considered.

### Limitations

The study was carried out at a single facility with patients from a small geographic area and backgrounds, which may have limited the sample size and generalizability of the findings. Additionally, the study was retrospective and had a follow-up time of only three years, which may not have been long enough to detect any differences in outcomes that could occur with a longer latency period.

## Conclusion

In patients with ULMCAD, IVUS-guided SI may result in better 3-year results than angiography-guided SI. Moreover, MSD could be an independent protective factor against cardiac mortality. However, these findings require further validation with multi-center studies and larger sample sizes.

## Data Availability

No datasets were generated or analysed during the current study.
